# Safety assesment of *Bacillus clausii* UBBC07, a spore forming probiotic

**DOI:** 10.1016/j.toxrep.2016.12.004

**Published:** 2017-01-05

**Authors:** Suvarna G. Lakshmi, N. Jayanthi, M. Saravanan, M. Sudha Ratna

**Affiliations:** a Centre for Research and Development, Unique Biotech Limited, Hyderabad, India; b Vipragen Biosciences Pvt. Limited, Mysore, India

**Keywords:** NOAEL, no observed adverse effect level, *B. clausii* UBBC07, *Bacillus clausii* UBBC07, Hgb, hemoglobin, Hct, hematocrit, RBC, red blood cell, WBC, white blood cell, RET, reticulocyte, MCV, mean corpuscular volume, MCH, mean corpuscular hemoglobin, MCHC, mean corpuscular hemoglobin concentration, ALT, alanine aminotransferase, AST, aspartate aminotransferase, TP, total protein, Alb, albumin, ALT, alanine amino transferase, Glu, glucose, T.chol, total cholesterol, Creat, creatinine, ALP, alanine amino phosphate, T.Bil, total bilirubin, Glob, globulin, Trig, triglycerides, BUN, blood urea nitrogen, Vol, volume, SG, specific gravity, Pro, protein, Leu, leucocytes, Ery, erythrocytes, EC, epithelial cells, C, casts, GC, granular casts, Cry, crystals, TPC, triple phosphate crystals, Acute toxicity, Subacute toxicity, NOAEL, *Bacillus clausii* UBBC07, Whole genome

## Abstract

Probiotics are vital bacteria that colonize the intestine and modify its microflora with benefits for the host. Very few members of the Bacillus group are recognized as safe for use and hence only a few strains are available as commercial preparations for application in humans and animals. Acute and subacute studies in rats were conducted to establish safety of *Bacillus clausii* (*B. clausii*) UBBC07. In the acute toxicity study, the oral LD50 for *B. clausii* UBBC07 was found to be >5000 mg/kg (630 billion cfu/kg) body weight. The NOAEL for *B. clausii* UBBC07 was found to be 1000 (126 billion cfu) mg/kg body weight/day by oral route in the subacute toxicity study. There were no significant differences between control and treated groups in any of the endpoints assessed using an OECD443 or OECD407 protocol.

*B. clausii* UBBC07 was found to be resistant to three antibiotics −clindamycin, erythromycin and chloramphenicol. Analysis of the whole genome sequence of *B. clausii* UBBC07 revealed that the antibiotic resistance genes are present in chromosomal DNA which is intrinsic and not transferable. Toxin genes were also found to be absent. These results suggest consumption of *B. clausii* UBBC07 is safe for humans.

## 1 Introduction

The popularity of probiotics has expanded exponentially recently, but along with their increased use, debate rages on how probiotics should be regulated and whether probiotics should be considered as a medical food, drug or a food supplement. Probiotics are defined as live microorganisms which, when administered in adequate amounts, confer a health benefit on the host [1]. Bacterial spore formers, mostly of the genus Bacillus constitute a major probiotic product in use today. They have to be adequately characterized for content, stability, and health effects, to be categorized as probiotics. Bacilli being ubiquitous consistently enter the gastrointestinal and respiratory tracts of healthy people through food, water and air. They have been isolated from gut and can reach upto 10^7^ cfu/g [2] and hence are considered to be one of the dominant components of the normal gut microflora. *Bacillus* strains offer some advantages over the more common *Lactobacillus* products in that they can be stored indefinitely in a desiccated form [3] without any deleterious effect on viability. In addition, they can also survive the low pH of the gastric barrier [4]. Bacillus species exert their beneficial effects, through immunomodulation i.e. induction of cytokines, competitive exclusion of gastrointestinal pathogens by competing for adhesion sites and secretion of antimicrobial compounds [5], [6]. Potential benefits of Bacillus sps include improved nutrition and growth, enhanced immunity and prevention of various gastrointestinal disorders (diarrhea, irritable bowel syndrome (IBS), inflammatory bowel disease (IBD), Crohn's disease, ulcerative colitis, necrotizing enterocolitis), respiratory disorders, allergies, skin disorders, bacterial vaginosis and cancer [7].

The production and use of probiotics has increased worldwide. As probiotics are strain specific, toxicity studies need to be carried out in order to establish safety. Though probiotics are Generally Regarded as Safe (GRAS), safety must not be taken for granted and every product must be evaluated on a case by case basis [8] Assessment of the acute and repeat dose toxicity must be carried out for all potential strains in order to establish safety. Lack of appropriate safety assessment can lead to probiotics being a source of food borne infectious disease and food poisoning as observed in a study for Bacillus cereus strain in China [9]. The FAO/WHO report has laid down a set of guidelines for a product to be used as a probiotic or novel supplement that must be judiciously followed [1], [10].

A few animal studies that include acute and sub-chronic toxicity testing as well as in vitro studies have been performed on some Bacillus species − *B. subtilis*, *B. indicus* [11], *B. coagulans* [12], [13] and *B. licheniformis* [14]. No adverse effects were reported in any of the studies. However, there are some bacillus species which were found to be pathogenic. Bacillus anthracis [15] being involved in systemic and hospital acquired infections and Bacillus cereus [16], [17] in diarrhea and food poisoning.

Widespread use of probiotic bacteria in conjunction and in close association with antibiotic use or rather misuse, can over time establish a reservoir of antibiotic resistant genes in probiotic bacteria. While intrinsic antibiotic resistance can be a desirable trait as probiotics help restore host gut microflora during a course of antibiotics, the transfer of resistant genes to humans offers serious clinical threats [18], [19].

*Bacillus clausii* (*B. clausii*) UBBC07 has been isolated by Unique Biotech Ltd, India. It is well characterized and has been deposited in MTCC (Microbial Type Culture Collection), India under Indian Patent deposit with the number MTCC 5472. *B. clausii* UBBC07 exhibits probiotic properties and being a spore forming probiotic, is stable over a wide range of temperatures with potential applications in a variety of formulations and foods. It was hence of interest to confirm its safety as time and again emphasis is laid on the importance of strain specificity in probiotics. Bacterial pathogenicity is strain specific and hence every bacterial strain promising as a probiotic should be tested individually [20].

Safety assessment of *B. clausii* UBBC07 was carried out through (a) acute and subacute oral toxicity studies in rats, (b) whole genome analysis and screening for absence of toxin genes and (c) establishing antibiotic resistance is intrinsic and not transferable.

## 2 Material and methods

### 2.1 Acute and subacute oral toxicity

#### 2.1.1 Test material

*Bacillus clausii* UBBC07 (MTCC no5472).

Reverse osmosis water was used as a vehicle for formulation preparation.

#### 2.1.2 Test animals

Rats of Sprague-Dawley (SD) strain of either sex with adequate fresh air supply (air changes 12–15 per hour), room temperature 22 ± 3 °C, relative humidity 30–70%, with a 12 h light and 12 h dark cycle. Three animals of same group were housed per cage in standard polycarbonate individually ventilated cages (size: L 430 × B 270 × H 150 mm) with stainless steel mesh top grill having facilities for holding pelleted food and drinking water. They were provided with pellets of animal food manufactured by M/s Ryan’s Biotechnologies Private Limited, Hyderabad, India. Autoclaved reverse osmosis water was provided ad libitum throughout the acclimatization and experimental period. Veterinary examination of all the animals was performed prior to administration of test item.

### 2.2 Method

#### 2.2.1 Acute toxicity

The study was performed according to the United States FDA Redbook, 2000 (Chapter IV- Guidelines for Toxicity tests), as well as the OECD guideline (423, sequential three steps). Each of three male (180.04 − 207.89 g weight) and three female (174.40–190.90 g weight) Sprague Dawley rats aged 11–12 weeks were treated with *B. clausii* UBBC07 or vehicle by oral gavage administration. There were three groups − *B. clausii* treated groups (Step 1 and Step II) and vehicle treated group (reverse osmosis water)

The administration volume was 10 mL/kg body weight. The animals were dosed using oral gavage feeding tubes.

In Step 1, three male and three female rats received a single dose of *B. clausii* UBBC07 by oral gavage administration at 5000 mg/kg body weight. No mortality was observed in the animals throughout the experimental period. As mortality was not observed in any animal dosed at 5000 mg/kg body weight, three naïve male and female were again treated with 5000 mg/kg body weight (Step II). As there was no mortality again, no further testing with another group (Step III) was required.

Vehicle Step received reverse Osmosis water only.

All animals were observed for effects on the respiratory, circulatory, autonomic & central nervous systems, skin, fur, eyes, mucous membranes, occurrence of secretions and excretions including stool consistency. Clinical signs of toxicity and mortality were observed once during the first 30 min and at approximately 1hr, 2hr, 3hr and 4hr on Day 1 following administration of test item and thereafter once daily during the 15 day observation period.

#### 2.2.2 Subacute toxicity

A repeated Dose 28-day Toxicity Study was carried out as per guideline OECD 407 to assess the systemic toxic potential of *B. clausii* UBBC07. This study was carried out after initial information on toxicity had been obtained by acute toxicity testing. A 28 day study provides information on the effects of repeated oral exposure. The highest dose used in the study was 1000 mg/kg body weight as this is the limit dose specified in the guidelines.

Grouping of animals was done by body weight stratification and randomization on computer-generated randomization procedure. The rats were distributed in 6 groups, each having 5 males and 5 females (Table 1a). The main groups were G1–Vehicle control (Reverse osmosis water), G2 − Low dose (100 (12.6 billion cfu) mg/kg body weight/day), G3 − Mid dose, (500 (63 billion cfu) mg/kg body weight/day) G4 − High dose (1000 (126 billion cfu) mg/kg body weight/day) Recovery groups (Table 1b) were G1R − Vehicle control recovery and G4R − High dose recovery. Rats aged 7–8 weeks with an average weight of 98.96–121.43 g for males and 90.50–120.07 g for females were selected for the study. The variation in the weight of rats was less than ± 20% of the mean body weight in each sex and group at the commencement of treatment. Rats were acclimatized for six days before the treatment. The dose formulations were administered once daily at approximately the same time each day (varying by ±2 h from the first day of administration) for 28 consecutive days using a dose volume of 10 ml. The different doses of test item formulations for low, mid, high and high dose recovery groups were prepared with the vehicle based on the weekly mean body weight of the animals. Recovery groups of Vehicle Control and High Dose groups were maintained for both male and female rats for further 14 days after the 28 day period without administering the test item or vehicle.

**Table 1a:** Main Groups- Repeated Dose 28 days Toxicity Study.

Group No.	Treatment Group	Dose (mg/kg B.wt/Day)	Dose Volume (mL/kg)	Conc. (mg/mL)	No. of rats	Sex
G1	Vehicle Control	0	10	0	5	M
					5	F
G2	Low dose	100	10	10	5	M
					5	F
G3	Mid dose	500	10	50	5	M
					5	F
G4	High dose	1000	10	100	5	M
					5	F

**Table 1b:** Recovery Groups- Repeated Dose 28 days Toxicity Study.

Group No.	Treatment Group	Dose(mg/kg B.wt/Day)	Dose Volume (mL/kg)	Conc. (mg/mL)	No. of rats	Sex
G1R	Vehicle Control	0	10	0	5	M
					5	F
G4R	High dose	1000	10	100	5	M
					5	F

The animals were observed for clinical signs once during acclimatization period. On the first three days of dosing, post dose observations were recorded twice at approximately hourly intervals for the first two hours. Subsequent post dose observations were recorded once every hour. Observations included changes in skin, fur, eyes, mucous membranes, occurrence of secretions and excretions and autonomic activity such as lacrimation, piloerection and unusual respiratory pattern. Changes in gait, posture and response to handling as well as the presence of clonic or tonic movements, stereotypies such as excessive grooming, repetitive circling and bizarre behavior such as self-mutilation & walking backwards were observed along with other clinical signs and were recorded. Animals were checked once daily for clinical signs during recovery period and on the day of necropsy. During fourth week of dosing period, sensory reactivity to stimuli such as auditory stimuli, visual stimuli (corneal reflex and pupil reflex) and proprioceptive stimuli were checked by using standard technique.

Blood samples for hematology and clinical biochemistry and urine samples were collected from all the animals after an overnight fasting. Blood parameters were analyzed using an ABC Vet hematology analyzer (scil Vet ABC TM Hematology Analyzer, scil Animal care company, USA). Blood smears were prepared by using standard techniques and stained with Giemsa stain for enumerating the cells. The white cell counts were expressed as percentage. Blood clotting time (seconds) was estimated by standard capillary tube method [21]. The clinical chemistry parameters were analyzed using COBAS C111 clinical chemistry analyzer (Roche Diagnostics, Basel, Switzerland). Electrolyte parameters were analyzed using a COBAS Roche 9180 electrolyte analyzer (Roche Diagnostics, Basel, Switzerland) and the urine parameters were analyzed using a COBAS 411 urine analyzer (Roche Diagnostics, Basel, Switzerland).

Following blood collection, rats were euthanized by carbon dioxide asphyxiation. Main and recovery study animals were euthanized rotating across dose groups such that similar numbers of animals from each group, including controls were necropsied throughout the day. Main and recovery study animals were subjected to a complete necropsy examination, which included evaluation of external and internal gross necropsy observations. Gross pathological examination was made and recorded. The organs were trimmed of any adherent tissue and weighed wet as soon as possible to avoid drying. Relative organ weights were calculated against fasting body weight. Paired organs were weighed together. The tissues were collected from all the animals and were preserved in 10% neutral buffered formalin. Additional tissue samples were collected for elucidation of abnormal findings.

Histopathological examination was carried out on the preserved organs of vehicle control (G1) and high dose groups (G4) rats. In addition, all gross lesions from all the rats were examined microscopically. The tissues were processed for routine paraffin embedding and 4–5 μm sections were stained with Mayer’s Haematoxylin and Eosin stain. Unused tissues were archived.

#### 2.2.3 Hemolysis and lecithinase activity

To check hemolysis property of *B. clausii* UBBC07, 5% sheep blood plates were prepared and spotted with *B. clausii* UBBC07 culture and incubated at 37 °C for 24–48 h. Lecithinase activity was checked by spotting *B. clausii* UBBC07 culture on plates containing *B. cereus* selective agar with egg yolk and polymyxin. To confirm the result, positive control *B. cereus* was taken and spotted like *B. clausii* UBBC07.

#### 2.2.4 Antibiotic resistance analysis

The antibiotic resistance pattern of *B. clausii* UBBC07 was identified by using the microdilution method according to EFSA 2012 guidelines [22]. Exponential phase cells of *B. clausii* UBBC07 were inoculated along with antibiotics at different concentrations. After incubation at 37 °C for 48 h, optical density was measured at 600 nm. Culture without antibiotics was used as control. The minimum inhibitory concentrations were used to determine resistance of *B. clausii* UBBC07 to the tested antibiotics.

#### 2.2.5 Genome analysis of *Bacillus clausii* UBBC07 for the absence of toxin genes and to establish antibiotic resistance is not transferable.

The whole genome sequence of *B. clausii* UBBC07 has been determined [23]. The whole-genome shotgun project has been deposited at DDBJ/ENA/GenBank under the accession no. LATY00000000.

#### 2.2.6 Whole genome sequence of *B. clausii* UBBC07 was analyzed in order to ensure safety.

Screening for known toxin genes in the whole genome of *B. clausii* UBBC07 was done − diarrheal enterotoxin *bceT*, haemolytic enterotoxin operon (hbl genes-*Hemolysin hblA,* hblC, hblD), non-haemolytic enterotoxin operon (nheABC genes- nehA, nehB, nehC, cytotoxin K (cytK),enterotoxin FM (entFM) and emetic toxin cereulide (cesB).

The genes were selected from a review of literature. Nucleotide sequences were downloaded from NCBI. Nucleotide blast was done using emetic nucleotide sequences as database and assembled contigs as query.

### 2.3 The Cutoff for significant BLAST hits was as follows

A minimum score value of 50, E-value of less than1 × 10^−8^, local matched region to be not less than 25% of the longer gene protein sequence and global matched region not less than 50% of the longer gene protein sequence.

### 2.4 Statistical analysis for toxicity studies in rats

All the results are expressed as Mean ± Standard Deviation (SD). The statistical analysis was carried out using a Statplus program. Data on the body weight, feed consumption, organ weights as well as clinical pathology data were analyzed. All the data was checked for normality with Shapiro-Wilk [24]. Data for each group of animals was subjected to analysis of variance (ANOVA). The treatment group animals were compared with control using *t*-test. Statistical significances of differences were calculated with one-way analysis of variance. All analyses and comparisons were evaluated at the 5% (P ≤ 0.05) level.

## 3 Results

### 3.1 Acute oral toxicity study in rats

The acute oral toxicity indicates that the lethal dose of *B. clausii* UBBC07 after single oral administration to male and female rats was greater than 5000 mg/kg (630 billion cfu/kg) body weight. All the animals appeared normal throughout the acclimatization period. There were no treatment-related variations in the mean body weight and net body weight gain. Neither any systemic (or) local toxicity were observed. There were no treatment related changes either in clinical signs or basic observations of autonomic activity. Macroscopic findings recorded at necropsy were also normal.

According to the Global Harmonized Classification System (GHS) the LD50 cut off for *B. clausii* after single oral administration rats was > 5000 mg/kg body weight, which is GHS category- 5. Criteria for Category 5 are intended to enable the identification of substances which are of relatively low acute toxicity hazard.

### 3.2 Subacute toxicity study in rats

In the subacute toxicity study, no mortality was observed and all rats appeared normal, without showing any signs or symptoms of abnormality at doses upto 1000 mg/kg/day (126 billion cfu) by the oral route of administration for 28 days. No significant effect on general health, body weight, food consumption, hematological or clinical chemistry profile or urine parameters was found. Relative organ weight and histological observations of vital organs in all treated group were unaffected. The No Observed Adverse Effect Level (NOAEL) is 1000 (126 billion cfu) mg/kg body weight/day for the test item, *B. clausii* UBBC07 on repeated 28 consecutive oral administrations to Sprague Dawley rats. Recovery group data is not provided as there were no significant changes between the main groups and placebo.

### 3.3 Hematology

After the administration of various doses to rats, there was no statistically significant effect on different hematological parameters like Hb, MCV, MCH, MCHC,clotting time (Tables 2a & 2b) as well as hematocrit, erythrocytes, leucocytes count and differential count (Tables 3a & 3b), in comparison to respective control animals.

**Table 2a:** Hematology parameters in Male rats on Day 29.

Hematology-Males										
Parameters										
Groups	Hgb (g/dl)	Hct (%)	RBC (10^6^ cells /mm^3^)	WBC (10^3^ cells /mm^3^)	RET (%)	MCV (μm^3^)	MCH (pg)	MCHC (g/dl)	Plt (10^6^cells /mm^3^)	Clotting Time (sec)
G1 Vehicle Control	14.0 ± 2.98	39.60 ± 10.79	6.81 ± 1.96	7.37 ± 1.45	3.8 ± 1.48	58.40 ± 1.52	21.1 ± 3.62	36.08 ± 6.20	655.20 ± 255.31	66.00 ± 27.25
G2 Low Dose	14.62 ± 2.69	38.54 ± 15.96	6.78 ± 3.04	9.00 ± 49	3.4 ± 1.14	58.6 ± 5.86	28.5 ± 22.39	46.30 ± 29.41	623.80 ± 145.37	54.00 ± 13.42
G3 Mid Dose	15.46 ± 3.31	36.88 ± 21.90	6.41 ± 4.27	7.50 ± 2.60	2.2 ± 1.48	63.00 ± 10.70	39.9 ± 32.63	58.32 ± 37.74	557.00 ± 121.20	57.00 ± 24.65
G4 High Dose	15.18 ± 1.88	36.26 ± 18.59	6.41 ± 3.55	8.44 ± 2.41	3.4 ± 1.14	59.40 ± 8.88	36.2 ± 32.62	56.36 ± 39.63	635.00 ± 211.26	66.00 ± 17.10

Key: n = 5; Values are in Mean ± SD; No statistically significant difference between groups.

Hgb- Hemoglobin; Hct- hematocrit; RBC- Red blood cell; WBC- White blood cell; RET- Reticulocyte; MCV- Mean corpuscular volume; MCH- Mean corpuscular hemoglobin; MCHC- Mean corpuscular hemoglobin concentration.

**Table 2b:** Hematology parameters in Female rats on Day 29.

Hematology-Females										
Parameters										
Groups	Hgb (g/dl)	Hct (%)	RBC (10^6^ cells /mm^3^)	WBC (10^3^ cells /mm^3^)	RET (%)	MCV (μm^3^)	MCH (pg)	MCHC (g/dl)	Plt (10^6^cells /mm^3^)	Clotting Time (sec)
G1 Vehicle Control	14.08 ± 1.63	41.91 ± 5.12	7.23 ± 1.06	6.42 ± 1.81	2.00 ± 0.55	58.20 ± 3.27	19.56 ± 1.32	33.62 ± 0.53	542.20 ± 139.31	48.00 ± 24.65
G2 Low Dose	15.60 ± 3.76	46.00 ± 12.73	7.91 ± 2.14	6.04 ± 1.92	4.20 ± 1.92	58.80 ± 4.21	20.16 ± 1.06	34.36 ± 2.05	449.80 ± 110.32	63.00 ± 19.56
G3 Mid Dose	16.54 ± 2.57	35.02 ± 20.67	6.01 ± 4.04	8.20 ± 2.07	2.60 ± 1.82	64.00 ± 11.58	46.22 ± 40.42	66.26 ± 40.70	467.00 ± 224.60	51.00 ± 13.42
G4 High Dose	15.34 ± 2.32	35.28 ± 16.75	5.72 ± 2.92	8.36 ± 1.57	2.40 ± 1.14	63.00 ± 4.74	33.64 ± 18.08	52.46 ± 25.55	551.00 ± 227.85	57.00 ± 19.56

Key: n = 5; Values are in Mean ± SD; No statistically significant difference between groups.

**Table 3a:** Differential count in Male rats on Day29.

Hematology					
Parameters					
Groups	Lymphocytes (%)	Monocytes (%)	Neutrophils (%)	Eosinophils (%)	Basophils (%)
G1 Vehicle Control	37.80 ± 12.81	11.60 ± 2.88	47.60 ± 14.88	3.00 ± 1.00	0.00 ± 0.00
G2 Low Dose	42.20 ± 6.61	14.40 ± 2.07	40.40 ± 6.73	3.00 ± 1.22	0.00 ± 0.00
G3 Mid Dose	40.60 ± 9.81	11.40 ± 2.30	45.80 ± 11.17	2.20 ± 0.84	0.00 ± 0.00
G4 High Dose	41.40 ± 13.07	11.20 ± 1.92	44.80 ± 13.97	2.60 ± 0.89	0.00 ± 0.00

Key: n = 5; Values are in Mean ± SD; No statistically significant difference between groups.

**Table 3b:** Differential count in Female rats on Day 29.

Hematology-Females					
Parameters					
Groups	Lymphocytes (%)	Monocytes (%)	Neutrophils (%)	Eosinophils (%)	Basophils (%)
G1 Vehicle Control	38.80 ± 2.86	13.40 ± 0.55	45.20 ± 3.56	2.60 ± 0.89	0.00 ± 0.00
G2 Low Dose	42.80 ± 6.26	10.80 ± 1.10	42.20 ± 6.83	4.20 ± 1.48	0.00 ± 0.00
G3 Mid Dose	42.20 ± 6.98	10.60 ± 1.52	44.40 ± 7.20	2.80 ± 0.84	0.00 ± 0.00
G4 High Dose	44.00 ± 5.00	9.60 ± 1.82	43.40 ± 6.58	3.00 ± 1.41	0.00 ± 0.00

Key: n = 5; Values are in Mean ± SD; No statistically significant difference between groups.

### 3.4 Clinical chemistry

The blood chemistry parameters such as total protein, alanine aminotransferase (ALT), aspartate aminotransferase (AST), alkaline phosphatase, urea nitrogen, total bilirubin and creatinine were not significantly altered (Table 4a, Table 4b).

**Table 4a:** Clinical Chemistry parameters in Male rats on Day 29.

Clinical Chemistry-Males													
Parameters													
Groups	TP (g/L)	Alb (g/L)	ALT (U/L)	AST (U/L)	Glu (mg/dL)	T.Chol (mg/dL)	Creat (mg/dL)	Urea (mg/dL)	Alp (U/L)	T.Bi (mg/dL)	Glob (g/L)	Trig (mg/dL)	Bun (mg/dL)
G1 Vehicle Control	52.58 ± 12.07	31.77 ± 6.96	32.96 ± 6.12	93.88 ± 18.42	151.42 ± 25.58	45.59 ± 7.99	0.20 ± 0.04	46.96 ± 5.65	221.82 ± 75.37	0.04 ± 0.04	20.81 ± 7.60	67.48 ± 31.32	21.92 ± 2.64
G2 Low Dose	60.58 ± 13.55	33.23 ± 8.83	34.44 ± 12.75	95.80 ± 18.30	139.81 ± 43.74	51.38 ± 19.70	0.23 ± 0.03	41.25 ± 13.75	223.04 ± 71.38	0.08 ± 0.04	27.35 ± 7.80	43.55 ± 11.26	19.25 ± 6.42
G3 Mid Dose	61.24 ± 3.81	35.16 ± 4.17	39.42 ± 11.86	105.32 ± 25.76	149.42 ± 30.61	55.53 ± 19.47	0.24 ± 0.06	33.51 ± 3.27	280.22 ± 82.80	0.07 ± 0.05	26.08 ± 6.46	46.57 ± 27.19	15.64 ± 1.53
G4 High Dose	50.00 ± 6.49	29.77 ± 7.27	32.00 ± 5.84	82.54 ± 16.63	140.49 ± 31.78	44.27 ± 5.24	0.22 ± 0.09	38.91 ± 11.20	223.56 ± 50.40	0.08 ± 0.06	20.23 ± 3.68	34.95 ± 13.39	18.16 ± 5.23

Key: n = 5; Values are in Mean ± SD; No statistically significant difference between groups.

TP- Total protein; Alb-Albumin; ALT- Alanine amino transferase; Glu-Glucose; T.chol- Total cholesterol; Creat- Creatinine; Alp- Alanine amino phosphate; T.Bi- Total bilirubin; Glob- Globulin; Trig- Triglycerides; BUN- Blood urea nitrogen.

**Table 4b:** Clinical Chemistry parameters in Female rats on Day 29.

Clinical Chemistry-Females													
Parameters													
Groups	TP (g/L)	Alb (g/L)	ALT (U/L)	AST (U/L)	Glu (mg/dL)	T.Chol (mg/dL)	Creat (mg/dL)	Urea (mg/dL)	Alp (U/L)	T.Bi (mg/dL)	Glob (g/L)	Trig (mg/dL)	Bun (mg/dL)
G1 Vehicle Control	55.12 ± 9.30	32.69 ± 5.28	30.89 ± 6.47	96.12 ± 9.80	107.53 ± 28.83	55.66 ± 16.39	0.23 ± 0.02	37.69 ± 9.30	191.88 ± 98.31	0.05 ± 0.03	22.43 ± 4.18	49.73 ± 29.01	17.59 ± 4.34
G2 Low Dose	61.72 ± 6.45	40.96 ± 6.69	28.56 ± 7.19	95.74 ± 22.14	151.21 ± 23.71	60.78 ± 6.39	0.29 ± 0.08	38.92 ± 6.34	118.20 ± 29.01	0.08 ± 0.07	20.76 ±	37.45 ± 7.56	18.16 ± 2.96
G3 Mid Dose	54.82 ± 8.94	35.10 ± 5.29	81.62 ± 20.00	81.62 ± 20.00	134.40 ± 20.62	45.49 ± 15.27	0.28 ± 0.10	41.18 ± 4.99	121.88 ± 42.09	0.06 ± 0.04	19.72 ± 3.92	39.53 ± 28.39	19.22 ± 2.33
G4 High Dose	58.44 ± 5.73	36.31 ± 4.94	38.22 ± 4.15	145.75 ± 31.84	145.75 ± 31.84	53.80 ± 7.61	0.28 ± 0.02	45.98 ± 6.58	149.00 ± 38.23	0.04 ± 0.03	22.13 ± 3.75	35.97 ± 7.57	21.46 ± 3.07

Key: n = 5; Values are in Mean ± SD; No statistically significant difference between groups

### 3.5 Urine analysis

The urine parameters for male and female rats were unaffected at different dosages of *B. clausii* UBBC07 (Tables 5a & 5b)

**Table 5a:** Chemical analysis of Urine in Males on Day-29.

Groups	Appearance	Vol (ml)	SG	pH	Pro (mg/dl)	Glu (mg/dl)	Leu (μl)	Ery (μl)	EC	C	GC	Cry	TPC
G1 Vehicle Control	Clear to turbid	6.64 ± 1.20	1.02 ± 0.00	7.70 ± 1.40	75.00 ± 0.00	0.00 ± 0.00	81.25 ± 37.5	11.00 ± 8.94	2.40 ± 1.14	0.80 ± 0.84	0.20 ± 0.45	3.20 ± 1.92	1.60 ± 1.82
G2 Low Dose	Clear to turbid	7.08 ± 0.90	1.02 ± 0.00	8.20 ± 1.10	75.00 ± 0.00	0.00 ± 0.00	100.00 ± 0.00	6.7 ± 5.8	1.60 ± 1.14	0.5 ± 0.48	0.00 ± 0.00	2.60 ± 1.52	1.80 ± 1.30
G3 Mid Dose	Clear to turbid	7.28 ± 0.59	1.02 ± 0.01	7.40 ± 1.34	70.00 ± 51.23	00.00 ± 0.00	100.00 ± 0.00	35.00 ± 22.36	1.80 ± 1.48	0.40 ± 0.89	0.20 ± 0.45	4.60 ± 2.88	1.40 ± 0.55
G4 High Dose	Clear to turbid	6.74 ± 0.65	1.01 ± 0.00	7.60 ± 0.55	45.00 ± 27.39	00.00 ± 0.00	75.00 ± 43.3	37.5 ± 14.4	1.60 ± 0.55	0.40 ± 0.55	0.00 ± 0.00	4.60 ± 2.88	1.00 ± 1.22

Key: n = 5; Values are in Mean ± SD; No statistically significant difference between groups.

Vol- Volume; SG- Specific gravity; Pro- Protein; Glu- Glucose; Leu- Leucocytes; Ery- Erythrocytes; EC- Epithelial cells; C- Casts; GC- Granular Casts; Cry- Crystals; TPC- Triple phosphate crystals.

EC:/HPF HPF:High Power Field.

C, GC, Cry &TPC;LPF.

LPF:Low Power Field.

**Table 5b:** Chemical analysis of Urine in Females on Day-29.

Urine Records − Females													
Parameters													
Groups	Appearance	Vol (ml)	SG	pH	Pro (mg/dl)	Glu (mg/dl)	Leu (ul)	Ery (ul)	EC	C	GC	Cry	TPC
G1 Vehicle Control	Clear to turbid	7.84 ± 1.38	1.01 ± 0.00	8.20 ± 0.45	75.00 ± 0.00	0.00 ± 0.00	100.00 ± 0.00	25.00 ± 4.50	2.00 ± 1.00	0.40 ± 0.55	0.00 ± 0.00	4.00 ± 4.18	1.00 ± 1.22
G2 Low Dose	Clear to turbid	7.36 ± 1.62	1.02 ± 0.00	7.80 ± 1.64	75.00 ± 0.00	0.00 ± 0.00	85.00 ± 35.54	20.00 ± 7.39	2.20 ± 1.10	0.60 ± 0.45	0.20 ± 0.45	2.60 ± 1.52	0.60 ± 0.89
G3 Mid Dose	Clear to turbid	7.68 ± 0.66	1.02 ± 0.01	7.50 ± 1.22	75.00 ± 0.00	00.00 ± 0.00	100 ± 25.50	28.00 ± 4.47	2.60 ± 0.55	0.40 ± 0.55	0.20 ± 0.45	3.80 ± 2.28	1.20 ± 1.64
G4 High Dose	Clear to turbid	7.82 ± 0.77	1.01 ± 0.00	8.40 ± 0.55	65.00 ± 22.36	00.00 ± 0.00	100.00 ± 0.00	25.00 ± 0.00	2.20 ± 1.10	0.40 ± 0.25	0.20 ± 0.45	6.00 ± 1.87	1.60 ± 1.34

Key: n = 5; Values are in Mean ± SD; No statistically significant difference between groups.

### 3.6 Organ weights

There was no alteration in the relative weight of vital organs such as kidneys, adrenals, spleen, heart, thymus, brain, testes, epididymis and prostate with seminal vesicles (Tables 6a & 6b).

**Table 6a:** Relative organ weights (%) in Males on Day 29.

Groups	Liver	Kidneys	Adrenals	Spleen	Heart	Thymus	Brain	Testes	Epididy- mides	Prostate & SV
G1 VehicleControl	4.01 ± 0.23	0.71 ± 0.02	0.02 ± 0.00	0.59 ± 0.14	0.36 ± 0.08	0.38 ± 0.32	1.07 ± 0.35	1.21 ± 0.33	0.47 ± 0.21	0.38 ± ± 0.17
G2 Low Dose	3.59 ± 0.18	0.78 ± 0.04	0.05 ± 0.06	0.56 ± 0.10	0.40 ± 0.09	0.22 ± 0.03	0.95 ± 0.07	1.42 ± 0.20	0.50 ± 0.11	0.56 ± 0.23
G3 Mid Dose	3.57 ± 0.38	0.76 ± 0.06	0.02 ± 0.00	0.43 ± 0.07	0.38 ± 0.02	0.21 ± 0.06	0.90 ± 0.07	1.27 ± 0.18	0.44 ± 0.12	0.52 ± 0.21
G4 High Dose	3.71 ± 0.57	0.82 ± 0.14	0.03 ± 0.01	0.47 ± 0.10	0.38 ± 0.06	0.19 ± 0.05	0.98 ± 0.17	1.42 ± 0.05	0.65 ± 0.23	0.46 ± 0.13

Key n = 5; Values are in Mean ± SD; No statistically significant difference between groups.

Relative organ weights (%) = Organ weight (g)/body weight (g) *100.

**Table 6b:** Relative organ weights (g) in Females on Day 29.

Groups	Liver	Kidneys	Adrenals	Spleen	Heart	Thymus	Brain	Ovaries	Uterus
G1 Vehicle Control	4.03 ± 0.47	0.82 ± 0.14	0.03 ± 0.01	0.58 ± 0.11	0.38 ± 0.06	0.20 ± 0.03	1.04 ± 0.08	0.09 ± 0.02	0.39 ± 0.12
G2 Low Dose	3.63 ± 0.36	0.77 ± 0.11	0.08 ± 0.12	0.50 ± 0.11	0.40 ± 0.04	0.19 ± 0.05	1.04 ± 0.10	0.08 ± 0.02	0.29 ± 0.07
G3 Mid Dose	3.39 ± 0.63	0.70 ± 0.04	0.03 ± 0.01	0.58 ± 0.23	0.38 ± 0.08	0.20 ± 0.08	1.02 ± 0.04	0.09 ± 0.02	0.62 ± 0.17
G4 High Dose	2.99 ± 0.41	0.70 ± 0.08	0.03 ± 0.00	0.43 ± 0.10	0.37 ± 0.07	0.25 ± 0.07	1.00 ± 0.04	0.06 ± 0.02	0.37 ± 0.12

Key n = 5; Values are in Mean ± SD; No statistically significant difference between groups.

Relative organ weights (%) = Organ weight (g)/body weight (g) *100.

### 3.7 Pathology

No test material related gross necropsy observations or histopathological changes were observed which is consistent with observations recorded in blood chemistry parameters. Erythematosus left hepatic lobe was observed in one female rat in vehicle control group.

### 3.8 Haemolysis and lecithinase activity

Hemolysis activity: *B. clausii* UBBC07 culture was spotted on 5% sheep blood agar and even after 48 h of incubation, haemolysis did not occur on sheep blood agar. For lecithinase production, *B. clausii* UBBC07 was spotted on *B. cereus* selective agar with egg yolk, there was no precipitation around the colony even after 48 h of incubation indicating that *B. clausii* UBBC07 is lecithinase negative.

### 3.9 Antibiotic resistance analysis

Antibiotic resistance profile of *B. clausii* UBBC07 (Table 7) indicated it was sensitive to three antibiotics, clindamycin (with 320 μg/ml minimum inhibitory concentration (MIC)), erythromycin (10 μg/ml MIC) and chloramphenicol (16 μg/ml). Analysis of whole genome sequences of *B. clausii* UBBC07 revealed that, the antibiotic resistance genes are present in chromosomal DNA which is intrinsic and not transferable. Hence *B. clausii* UBBC07 cannot transfer its resistance to any other organism.

**Table 7:** Antibiotic sensitivity profile of *Bacillus clausii* UBBC-07.

S.No	Antibiotic	Break point MIC^a^ (μg/ml)	MIC for *B. clausii* UBBC-07(μg/ml)
1	Clindamycin	4	320
2	Erythromycin	4	10
3	Chloramphenicol	8	16

a MIC: Minimum Inhibitory Concentration.

### 3.10 Genome analysis for establishing antibiotic resistance is intrinsic

**Fig. 1:**
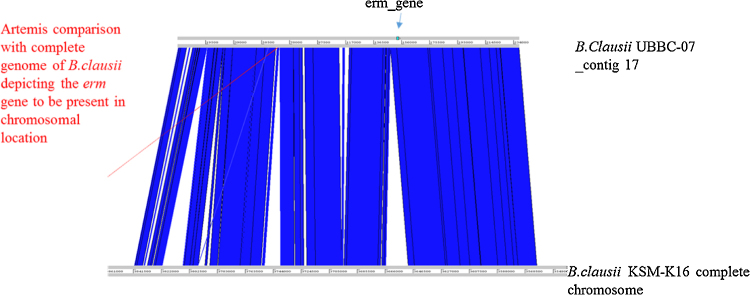
Artemis comparison (erm gene).

The erm34 gene from *B. clausii* DSM8716 was used to carry out blast against the genome of *B. clausii* UBBC07. The UBBC07 genome encodes erm gene with identity of 96% at nucleotide level (Fig. 1). The complete genome of *B. clausii* strain KSM-K16 is available in the public domain (Accession No. AP006627) and erm gene is encoded in the chromosome. Artemis comparison revealed that as in KSM-K16 genome, the erm gene is also chromosomally encoded in UBBC07 (Table 8). Further the GC content of the erm gene is similar as the GC content of *B. clausii* genome i.e. around 44% suggesting that gene is not horizontally transferred and is intrinsic and non-transferrable as it is chromosomally encoded.

**Table 8:** erm gene GC content and identity:.

Organism	GC content	Size	Coverage
*B. clausii* UBBC-07	44.63	4,197,324	241
*B. clausii*UBBC-7_contig 17	44.06	237,768	300
*B. clausii*-07_contig17_erm_gene	45.06		

	*B. clausii* DSM8716_erm34
Nucleotide sequences	
*B. clausii*-07_contig17_erm_gene	800/833 (96%)
Protein sequences	
*B. clausii*-07_contig17_erm_gene	272/281 (97%)

**Fig. 2:**
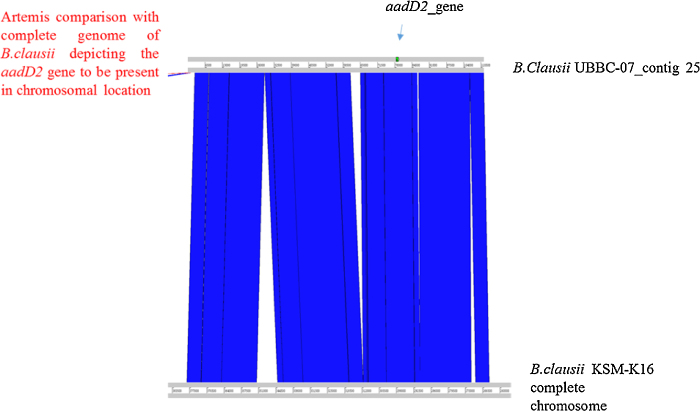
Artemis comparison (aadD2 gene).

Furthermore, the aadD2 gene sequence was used to carry out blast against the genome of *B. clausii* UBBC07. The UBBC07 genome was also found to encode aadD2 gene with identity of 98% at nucleotide level (Table 9). Artemis comparison revealed that like the erm gene, the aadD2 gene is also chromosomally encoded in UBBC07 (Fig. 2). Further the GC content of aadD2 gene is similar as the GC content of *B. clausii* genome i.e. around 44% suggesting that gene is not horizontally transferred and is intrinsic and non-transferrable as it is chromosomally encoded.

**Table 9:** aadD2 gene GC content and identity:.

Organism	GC content	Size	Coverage
*B. clausii* UBBC-07	44.63	4,197,324	241
*B. clausii* UBBC-07_contig25	44.06	111,547	338
*B. clausii* UBBC-07_contig25_aaD2 gene	44.42		

	*B. clausii* KSM-K16
Nucleotide sequences	
*B. clausii* UBBC-07_contig25_aaD2 gene	754/773(98%)
Protein sequences	
*B. clausii* UBBC-07_contig25_aaD2 gene	245/255(96%)

**Fig. 3:**
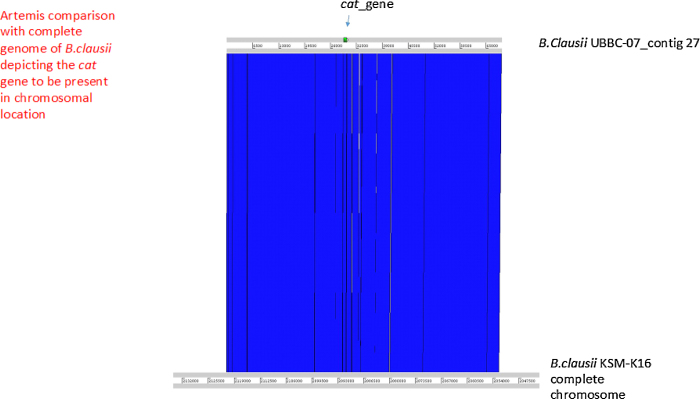
Artemis comparison (cat gene).

The cat gene sequence was used to carry out blast against the genome of *B. clausii* UBBC07. The UBBC07 genome was found to encode the cat gene with identity of 99% at nucleotide level (Table 10). Artemis comparison revealed that like the erm and aadD2 genes, the cat gene is also chromosomally encoded in UBBC07 (Fig. 3). The GC content of cat gene is lower (32%) as compared to the GC content of *B. clausii* genome i.e. around 44% suggesting that gene could have been acquired by horizontal gene transfer but still chromosomally encoded.

**Table 10:** *cat* gene GC content and identity.

Organism	GC content	Size	Coverage
*B. clausii* UBBC-07	44.63	4,197,324	241
*B. clausii* UBBC-07_contig27	43.92	68,962	140
*B. clausii* UBBC-07_contig27_cat_gene	32.1		

	*B. clausii* KSM-K16
Nucleotide sequences	
*B. clausii* UBBC-07_contig27_cate_gene	641/648(99%)
Protein sequences	
*B. clausii* UBBC-07_contig27_cate_gene	214/215(99%)

### 3.11 Screening for toxin genes

The *B. clausii* UBBC07 whole genome was used for the detection of toxin genes. The *bceT* gene, which encodes the single-component enterotoxin T, was not detected and the hemolytic enterotoxin *hbl*, which was found to carry three genes (*hblA, hblB* and *hblC*) was not found. Non-hemolytic enterotoxin (*Nhe*) which codes for all the three genes *(nheA, nheB* and *nheC*) was not observed in *B. clausii* UBBC07 (Table 11).

**Table 11:** Toxin genes.

Genes	*B. clausii*	
	BLASTIN	TBLASTIN
Diarrheal enterotoxin bceT	No significant hits	159/357(44%)
		E value:2e-075

Haemolytic enterotoxin operon (hbl genes)		
Hemolysin hblA	No significant hits	No significant hits
hblC	No significant hits	No significant hits
hblD	No significant hits	No significant hits

Non-haemolytic enetotoxin operon (nhe ABC genes)		
nhe A	No significant hits	No significant hits
nhe B	No significant hits	No significant hits
nhe C	No significant hits	No significant hits
Cytotoxin K (cytK)	No significant hits	No significant hits
Enterotoxin FM (entFM)	No significant hits	59/142(41%),e value 5e-027
Emetic Toxin Cereulide (cesB)	No significant hits	115/512 (22%) E value 1e-028

Cutoff for BLAST:. • A minimum score value of 50. • E-value of less than1 × 10^−8^. • local matched region to be not less than 25% of the longer gene protein sequence. • Global matched region not less than 50% of the longer gene protein sequence.

Blast search did not reveal any significant hits confirming the absence of any toxin genes.

## 4 Discussion

In the present study, we extensively investigated the safety of the probiotic strain *B. clausii* UBBC07. The acute and subacute toxicity studies in rats established that *B. clausii* UBBC07 is safe. Absence of any adverse effects of *B. clausii* UBBC07 indicated that this probiotic strain did not exhibit gross acute oral toxicity effects on the experimental animals, general health status, growth and development. Our results are in concordance with other similar studies advocating oral toxicity testing as a fundamental test for assessing safety of the test strain in animal models [25]. The OECD 423 and 407 guidelines according to which the present studies were performed provide a robust assessment of toxicity

The ‘No Observed Adverse Effect Level (NOAEL)’ derived from the repeated dose 28 day toxicity study was 1000 (126 billion cfu) mg/kg body weight/day. Since the concentration of *B. clausii* UBBC07 used was 126 × 10^9^ CFUs/g, this corresponds to 126 × 10^9^ CFUs/kg. For an average 70 kg human being, this corresponds to 88.2 × 10^11^ CFUs. Because the suggested human dose is in the range of 2 × 10^9^ to 6 × 10^9^ CFUs, this gives a safety factor ranging from 1470 to 4, 410 times.

Hemolytic and lecithinase activity is an indication of the presence of cytotoxic phospholipases that are associated with virulence of a given bacterial strain [26] Our data indicates that *B. clausii* UBBC07 does not produce lecithinase nor does it possess hemolytic activity corraborating further its safety.

Many strains of *B. clausii* are considered antibiotic resistant and hence they are recommended for use along with antibiotics [27], [28]. Antibiotic resistance is a not a safety issue as long as there is no risk of resistance transfer. Screening of whole genome sequence of the *B. clausii* UBBC07 revealed that the antibiotic resistance genes are present in chromosomal DNA which is intrinsic and not transferable. Hence *B. clausii* UBBC07 cannot transfer its resistance to any other organism. A review of literature indicated that other strains of *B. clausii* also encode genes for antibiotic resistance which are stable and non transferable. These genes include *erm* gene that encodes a ribosomol methylase protein [29], *cat* gene for production of chloramphenicol acetyltransferase and *aadD2* gene, responsible for the production of an aminoglycoside 4ʹ-O-nucleotidyltransferase [30], [31].

In addition, absence of toxin genes in the genome of *B. clausii* UBBC07 advocates the safety of the strain. Genes for the toxins *bce*T and *cyt*K were also absent. Our data is in accordance with the report of [32] in which 333 Bacillus strains outside the *B. cereus* group have been analyzed, and none of these strains produced *B. cereus*-like toxins.

Finally, the generation of the complete genetic makeup of *B. clausii* UBBC07 has revealed many beneficial probiotic traits that contribute to the safety of the strain for its use in a wide range of health-promoting applications [23].

## 5 Conclusion

Taken together, our data indicate that *B. clausii* UBBC07 is safe. The No Observed Adverse Effect Level (NOAEL) for *B. clausii* UBBC07 was found to be 1000 (126 billion cfu) mg/kg body weight/day by oral route. This was well tolerated and did not cause any lethality or toxic clinical symptoms in the experimental rats. *B. clausii* UBBC07 does not produce lecithinase and it is non-hemolytic. The absence of toxin genes and transferable antibiotic resistance genes further revealed that *B. clausii* UBBC07 is safe for human consumption as probiotic.

## Conflict of interest

The authors are manufacturers of *Bacillus clausii* UBBC07.

However, all the studies were independently carried out with no intervention from the manufacturers. The toxicity studies were conducted at Vipragen Biosciences Pvt. Ltd, India and whole genome sequencing and analyses was carried out at Institute of Microbial Technology (IMTECH), India.
